# Maize Bushy Stunt Phytoplasma Favors Its Spread by Changing Host Preference of the Insect Vector

**DOI:** 10.3390/insects11090600

**Published:** 2020-09-05

**Authors:** Anderson Ramos, Mariana Bossi Esteves, Mayerli Tatiana Borbón Cortés, João Roberto Spotti Lopes

**Affiliations:** Departamento de Entomologia e Acarologia, Escola Superior de Agricultura “Luiz de Queiroz” (ESALQ), Universidade de São Paulo (USP), Avenida Padua Dias, 11, Piracicaba 13418-900, Brazil; mbesteves1@gmail.com (M.B.E.); mtborbonc@usp.br (M.T.B.C.); jrslopes@usp.br (J.R.S.L.)

**Keywords:** corn leafhopper, corn stunt, phytopathogenic mollicute, vector manipulation

## Abstract

**Simple Summary:**

Phytopathogenic bacteria such as phytoplasmas induce physiological changes in their host plants that may modulate the behavior of an insect vector in favor of their own spread. In this study we investigate changes in the host selection behavior of the leafhopper vector, *Dalbulus maidis* (DeLong and Wolcott), in choice tests between healthy vs. maize bushy stunt phytoplasma (MBSP)-infected maize leaves (*Zea mays* L.), for insects previously exposed to infected plants (named “bacteriliferous”) or not (naive). The results showed that males and naive females of *D. maids* did not distinguish the treatments when infected leaves were still asymptomatic, whereas bacteriliferous females prefer to settle on healthy leaves, a behavior that favors pathogen inoculation and primary spread at early crop stages. During the symptomatic phase of maize infection, naive males and females were initially attracted to infected leaves, favoring pathogen acquisition; interestingly, the females tend to move towards healthy leaves a few hours later, a behavioral shift that promotes secondary spread. Overall, this study presents evidences that MBSP optimizes its spread in maize crops by influencing the host selection behavior of the leafhopper vector.

**Abstract:**

Plant pathogenic bacteria may influence vector behavior by inducing physiological changes in host plants, with implications for their spread. Here, we studied the effects of maize bushy stunt phytoplasma (MBSP) on the host selection behavior of the leafhopper vector, *Dalbulus maidis* (DeLong and Wolcott). Choice assays contrasting leaves of healthy (mock-inoculated) vs. infected maize (*Zea mays* L.) were conducted during the asymptomatic and symptomatic phases of plant infection, with leafhopper males or females previously exposed to infected plants (bacteriliferous insects) or not. In each assay, 40 adults were released in choice arenas where only the leaves of two plants from each treatment were offered and visible, and the insects landed on the leaves were counted 1, 2, 3, 5, 7, 9, 11 and 23 h after release. During the asymptomatic phase of plant infection, an effect was observed only on bacteriliferous females, who preferred leaves of healthy plants 5 h after release or later. The symptomatic phase triggered a pull–push effect on non-bacteriliferous females, who were first attracted to symptomatic leaves but hours later moved to healthy leaves. Non-bacteriliferous males initially preferred symptomatic leaves (up to 5 h after release) and later became equally distributed between treatments. Bacteriliferous males and females initially did not discriminate between healthy and symptomatic leaves, but only the females tended to move to healthy leaves 9 h after release. Oviposition was drastically reduced on symptomatic leaves. The changes in vector behavior induced by MBSP favor its primary spread, since bacteriliferous females prefer healthy leaves at early (asymptomatic) stages of the crop. At later stages, secondary spread may be favored because non-bacteriliferous females are initially attracted to infected (symptomatic) leaves, allowing pathogen acquisition and subsequent transmission as they move to healthy plants.

## 1. Introduction

Host selection, a process in which phytophagous insects seek suitable plants for feeding, colonization and/or oviposition, plays a fundamental role in the spread of phytopathogens transmitted by insect vectors [1,2]. Several plant cues influence this process. Before landing, the visual and olfactory stimuli provide cues for pre-alighting behavior and plant choice [1]. After landing, the tactile and gustatory cues guide the insect’s feeding behavior, indicating whether the plant is suitable for feeding and reproduction [1,3].

Most insects that transmit plant pathogens are hemipteroids (Hemiptera and Thysanoptera), with piercing–sucking mouthparts [4,5]. Interestingly, changes in the host selection behavior of these insect vectors can be induced by pathogen infection, which may be adaptive mechanisms of the pathogen for optimization of its own spread (“vector manipulation” hypothesis) [6]. These changes can be promoted directly, by the presence of the phytopathogen in the vector’s body, or indirectly, by physiological or morphological changes in the infected host plant [6,7,8].

Substantial alterations in plant characteristics induced by pathogen infection have been documented for phytoplasmas, which are wall-less, Gram-positive phytopathogenic bacteria in the class Mollicutes [9,10]. Phytoplasmas (“*Candidatus* (*Ca*.) Phytoplasma”) colonize the phloem of a wide variety of herbaceous and woody plants and are associated with numerous economically important diseases in crop plants [11]. They are naturally transmitted by phloem sap-feeding hemipterans, e.g., leafhoppers, planthoppers and psyllids, in a persistent propagative manner [5,12]. In this mode of transmission, the acquisition and inoculation of the pathogen usually requires relatively long periods (several minutes to hours) of feeding in the sieve tube elements of the host plant. After bacterial acquisition, the pathogen colonizes several internal organs of the insect, until it reaches the salivary glands and is inoculated into a new plant. There is a latent period between acquisition and inoculation with a duration of weeks [12].

Studies have shown that phytoplasmas can influence host plant selection of their vectors [12]. Plants of *Malus domestica* Borkh. (Rosaceae) infected with the apple proliferation phytoplasma (*Ca*. P. mali) were more attractive for feeding to newly emerged adults of the psyllid vector, *Cacopsylla picta* (Foerster) (Hemiptera: Psyllidae), due to the emission of the volatile compound β-caryophyllene [13,14]. Studies of the aster yellows phytoplasma strain witches’ broom (AY-WB) (*Ca*. P. asteris) detected the presence of several possible effector proteins. Two of them, named SAP11 and SAP54, had an effect on hormonal regulation, defense mechanisms and development of the host plant, *Arabidopsis thaliana* (L.) (Brassicaceae), which affected the biology and behavior of the leafhopper vector, *Macrosteles quadrilineatus* Forbes (Hemiptera: Cicadellidae). Transgenic plants expressing SAP11 or SAP54 attracted more leafhoppers than healthy non-transgenic plants but only SAP11 increased the fecundity of the leafhopper vector [15,16].

Maize (*Zea mays* L.) is a crop of worldwide importance that has been affected by severe epidemics of the maize bushy stunt phytoplasma (MBSP) (*Ca*. P. asteris) in several countries of the American continent [17]. MBSP induces symptoms of leaf discoloration (reddening or yellowing), stunting, lateral branching and ear proliferation, and is transmitted by the corn leafhopper *Dalbulus maidis* (DeLong and Wolcott) (Hemiptera: Cicadellidae), which is a monophagous insect specific to maize [18,19]. MBSP is closely related to AY-WB (both belong to Ca. P. asteris) and has an effector protein (SAP11_MBSP_) homologous to SAP11 of AY-WB, which is known to induce axillary branching and affect female inflorescence development in maize, but has no impact on the fecundity of *D. maidis* [10].

Despite the destructive potential of this phytoplasma on maize, there is limited information about the impact of diseased plants on host selection behavior of *D. maidis*. Garcia-Gonzalez et al. [20] reported the preference of *D. maidis* adults for settling and oviposition on infected plants during the asymptomatic phase, but this preference was reverted when MBSP symptoms developed. Because their study was performed by offering whole plants to the insects in choice assays, it is not clear whether the effect was due to differences in plant size between healthy and diseased plants, or to other morphological and physiological changes caused by the disease. It also remains unknown whether the gender of the insect vector or its infection with the phytoplasma is related to the choice.

The present study was designed to evaluate the effect of leafhopper gender and infection by MBSP on preference for settling and oviposition on leaves of healthy versus MBSP-infected maize plants, during the asymptomatic and symptomatic phases of the disease. It was also evaluated if the vector is able to acquire the MBSP during the relatively short period that it shows preference for symptomatic infected plants, and subsequently transmit the pathogen to healthy plants. This information is fundamental for a better understanding of the epidemiology of the disease and elaboration of control tactics.

## 2. Materials and Methods

### 2.1. Production of Healthy Leafhoppers and Maize Plants

The laboratory colony of *D. maidis* was established with insects collected from maize in the municipality of Jardinópolis, SP, Brazil (20.912931S; 47.896399W). The leafhopper colony was reared on maize plants, in aluminum frame cages (30 × 30 cm (base) by 50 cm (height)) covered with an anti-aphid mesh, as described by Oliveira et al. [21]. The rearing cages were maintained in a greenhouse equipped with a pad fan cooling system and a thermostat-activated heater for temperature control (25 ± 5 °C), under natural light.

Healthy maize seedlings of the hybrid 2B433PW (Brevant^®^ seeds, Corteva Agriscience, Wilmington, DE, USA) used in the leafhopper rearing and in the experiments were sown in 200-mL plastic pots (three to four seeds/pot) containing a substrate of pine bark, vermiculite, simple superphosphate and potassium nitrate (Tropstrato HT (Vida Verde, Mogi Mirim, SP, Brazil)). The seedlings were produced in a vector-proof screenhouse (anti-aphid mesh) and were fertilized three times a week as described by Esteves et al. [22].

In order to obtain a colony free of virus and mollicutes transmitted by *D. maidis*, about 200 adults from the original colony were allowed a 2-day oviposition period on three to four maize seedlings at stage V3-V4 (three to four fully expanded leaves). After 8 days, the eggs were excised from the leaves with a scalpel blade, under a stereoscopic microscope with 10× magnification (Model: SMZ-168-TL, Motic, Richmond, BC, Canada). The excised eggs were placed in Petri dishes containing a sheet of moistened filter paper, which was kept in an incubator at 25 ± 1 °C. The filter paper was humidified daily and the hatched nymphs were transferred to new healthy maize seedlings. The healthy emerged adults were multiplied inside rearing cages as described above.

### 2.2. Origin of the MBSP Isolate and Production of Infected Maize Plants and Leafhoppers

The MBSP isolate used in the experiments (named R4) was obtained from maize plants with typical symptoms of infection in Piracicaba, SP, Brazil [19]. This isolate was perpetuated in plants of the susceptible hybrid 2B433PW through leafhopper transmission. For isolate perpetuation and production of leafhoppers and plants for the experiments, the following procedure was established. Groups of 300–400 nymphs of 2nd and 3rd instars from the healthy colony of *D. maidis* were confined on maize plants infected with isolate R4 (source plants) for an acquisition access period (AAP) of 4 days. Then, the individuals were transferred to healthy maize plants for a latent period (LP) of approximately 25 days [21]. During the LP, the plants were changed twice a week to prevent the hatching of nymphs from eggs laid by emerging adults.

After the LP, the adults previously exposed to infected plants (herein named “bacteriliferous”) were used in the free-choice experiments or for the phytoplasma inoculation of new plants. Non-bacteriliferous adults from the same colony and generation, but only exposed to healthy plants under the same conditions, were also used in the experiments. Before the experiments, the insects were segregated by gender under a stereoscopic microscope (8× magnification); the insects were immobilized on a cold Petri dish with crushed ice underneath for genitalia analysis.

For the production of new source plants, the bacteriliferous insects were confined on healthy maize seedlings in stage V1 for an inoculation access period (IAP) of 4 days (15 insects/plant), inside cylindrical cages made of clear acetate (10 cm in diameter by 45 cm (height)) with two side openings of 4 cm in diameter and the top opening sealed with “voile” fabric for ventilation. The whole transmission process (AAP, LP and IAP) was conducted in a climate-controlled room at 25 ± 5 °C and a photophase of 14 h. The source plants were submitted to a new AAP for MBSP perpetuation when symptoms became apparent, around 45 days after the IAP.

The production of MBSP-infected and healthy (mock-inoculated) maize plants for the experiments followed the same procedure, except that maize seedlings in the V1 stage were exposed to bacteriliferous and non-bacteriliferous insects, respectively, during a 2-day IAP using 25 insects/plant. After the IAP, all plants (including the source plants) were sprayed with lambda-cyhalothrin (1 mL/L) (Karate Zeon 50 CS, Syngenta, Paulínia, SP, Brazil) plus mineral oil (4 mL/L) (Assist^®^ EC, BASF, Jaguariúna, SP, Brazil) for the elimination of leafhopper eggs and kept in a vector-proof screenhouse with an anti-aphid mesh.

### 2.3. Free-Choice Assays of D. maidis on Leaves of Healthy vs. Symptomatic or Asymptomatic MBSP-Infected Maize

A series of dual choice tests were conducted to evaluate the settling and oviposition preference of bacteriliferous or non-bacteriliferous males and females of *D. maidis* for leaves of healthy (mock-inoculated) vs. MBSP-infected plants of the same age, exhibiting symptoms (45–55 days after IAP) or not (15–16 days after IAP).

The experiments were conducted in a cubic arena (30 × 30 × 30 cm) (Figure S1). Each side of the arena was wrapped with white adhesive tape to prevent insects from viewing the plants in full, thus allowing the choice only by cues displayed by the leaves inside the arena. Two plants from each treatment had their leaves exposed through one of the side openings, one leaf from each plant per side, with the treatments distributed equidistantly and interspersed. The experimental arena was maintained throughout the test period in a room with a controlled climate (25 °C ± 1 °C, 14 h photophase), with a light source of 31.6 ± 0.6 mmol^−1^ (four fluorescent lamps TLD 32W/840NG EcoMASTER, Philips, Barueri, SP, Brazil), positioned 1.5 m above the arenas.

Independent experiments were conducted to assess the effect of leafhopper gender and infectivity for healthy (mock-inoculated) vs. MBSP-infected maize plants: (1) with asymptomatic plants (15–16 days after IAP) and (2) with symptomatic plants (45–55 days after the IAP). The experimental design of the free-choice assays was completely randomized with at least 10 replications per treatment (non-bacteriliferous or bacteriliferous) and for each gender. In each replication, 40 insects were released from a “Falcon” tube positioned at the top of the arena and the number of leafhoppers that landed on each leaf was recorded 1, 2, 3, 5, 7, 9, 11 and 23 h after release. At the end of the assay, the plants were kept in a vector-proof screenhouse with an anti-aphid mesh, and the eggs laid on the exposed leaves were counted 7 days later, under a stereoscopic microscope with 10× magnification. In the assays with asymptomatic plants, after counting the eggs, the plants were kept in the screenhouse until MBSP symptoms appeared to confirm the infection.

### 2.4. Acquisition and Transmission Efficiency from Symptomatic Plants with MBSP

The acquisition and transmission efficiency of *D. maidis* was analyzed due to the initial preference (up to 6 h after insect release in the arena) of non-bacteriliferous adults for plants with MBSP symptoms in the previous experiment. For this, newly emerged non-bacteriliferous insects (4–5 days after emergence) were caged on leaves of symptomatic plants for an AAP of 6 h. After the AAP, the insects were removed from the plants and kept on uninfected plants for a 25-day LP, during which the plants were replaced weekly. Afterwards, the insects were caged on six healthy maize seedlings at the V1 stage (25 insects/plant) for a 4-day IAP. Then, the insects were stored in absolute alcohol and kept in a freezer (−20 °C) for molecular detection of MBSP. To determine transmission, the inoculated seedlings were evaluated for MBSP symptoms 50 days after the IAP. During AAP and LP, the insects were confined on the plants in sleeve cages made of “voile” fabric. During the IAP, the insects were kept in the acetate cages described before. The AAP, LP and IAP were conducted in the same climate-controlled room described for the production of infected maize plants.

### 2.5. Detection of MBSP in D. maidis

The DNA of the insects used in the transmission experiments was extracted following the cetyltrimethylammonium bromide (CTAB) protocol described by Marzachì et al. [23]. The nucleic acid samples were analyzed by PCR using the Dream Taq PCR Master Mix (Thermo Scientific, São Paulo, SP, Brazil). The sets of primers P1 and AYint were designed from the 16 s region of the phytoplasma rDNA by Smart et al. [24]. Each reaction contained positive controls (DNA from plants that had already been diagnosed as positive in previous PCRs) and negative controls, one represented by Milli-Q^®^ water and the other by DNA from a healthy plant. PCR was performed in a PTC-100 thermocycler (MJ Research, Inc., Watertown, MA, USA) programmed with an initial cycle of 94 °C for 2 min, 35 denaturation cycles at 94 °C for 1 min, annealing at 56 °C for 1 min, followed by extension at 72 °C for 2 min and 1 cycle at 72 °C for 5 min. The amplified fragments were analyzed by electrophoresis on a 1% agarose gel. After electrophoresis, the gel was visualized on a Compact Digimage System, UVDI series transilluminator (Major Science, Saratoga, CA, USA).

### 2.6. Statistical Analysis

The data of the mean number of insects that settled on plants in the free-choice experiments with symptomatic and asymptomatic plants were submitted to analysis of variance (ANOVA) according to the repeated measures model. The data were tested for the model’s assumptions; therefore, normality and sphericity tests were performed. In cases where the data did not show sphericity, the Greenhouse–Geisser correction was used for comparisons. In some cases, the points used for the construction of the curve had no normality, so they were not considered in the statistical analysis. For the comparison of the curves, five to eight points were considered, depending on the number of points that had to be discarded due to lack of normality. A Student’s *t*-test was also performed to compare the number of insects on healthy vs. infected plants at two points (evaluation periods) within the curve; the points chosen were the first time period when all insects had left the releasing tube and the third point after stabilization of the curve (for the experiment with asymptomatic plants) or the second point after the inflection of the curve (experiment with symptomatic plants). The data points submitted to the Students’s *t*-test were tested for normality and homoscedasticity, and only one of the points did not show normality; the data were then transformed into Log(x) and the comparisons were performed. The comparison of oviposition data was performed using a Student’s *t*-test; when there was no normality, the data were transformed to Log(x). In cases in which even with the transformation of the data it was not possible to obtain normality, the Mann–Whitney non-parametric test was used. All statistical tests were performed using SPSS^®^ Statistics version 25 statistical software (IBM, New York, NY, USA).

## 3. Results

### 3.1. Preference of D. maidis for Healthy vs. Asymptomatic MBSP-Infected Maize Leaves

**Figure 1:**
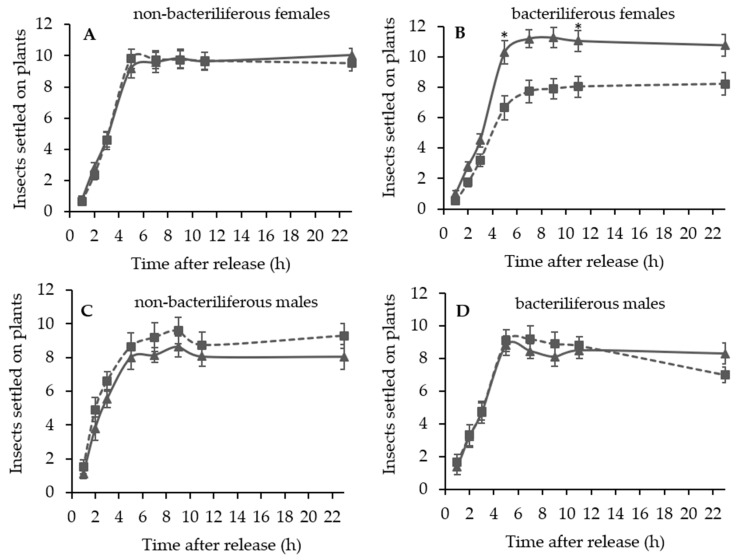
Average number (±SEM) of *Dalbulus maidis* adults on leaves of healthy “mock-inoculated” (solid line) vs. maize bushy stunt phytoplasma (MBSP)-infected asymptomatic (dotted line) maize plants, at successive time periods after insect release in the choice arena. (**A**) Non-bacteriliferous females; (**B**) Bacteriliferous females; (**C**) Non-bacteriliferous males; (**D**) Bacteriliferous males. Means at 5 and 11 h after release were compared by *t*-test and the asterisk (*) represents statistical difference (*p* < 0.05).

**Figure 2:**
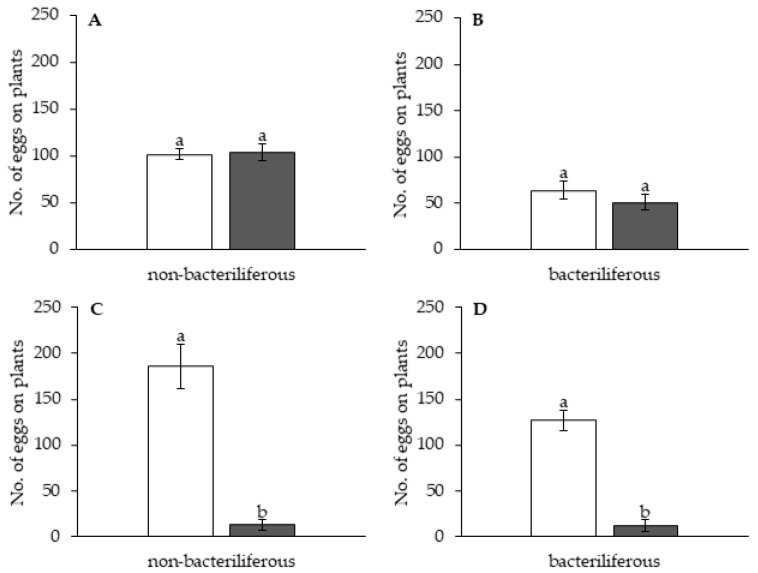
Average number of eggs (±SEM) laid by bacteriliferous and non-bacteriliferous *Dalbulus maidis* females on leaves of healthy “mock-inoculated” (white bars) vs. maize bushy stunt phytoplasma (MBSP)-infected (black bars) maize plants during 24-h choice assays. Infected plants were either in the asymptomatic (**A**,**B**) or symptomatic (**C**,**D**) phase of the disease. Means with the same lowercase letters are not statistically different by the *t*-test (**A**,**B**) or Kruskal–Wallis test (**C**,**D**) (*p* < 0.05).

In the free-choice assays contrasting leaves of healthy (“mock-inoculated”) vs. MBSP-infected maize plants during the asymptomatic stage of the disease, bacteriliferous females of *D. maidis* preferred to land and settle on leaves of healthy plants (F = 7.11; d.f. = 1;13; *p* = 0.019), whereas non-bacteriliferous females (F = 0.002; d.f. = 1, 14; *p* = 0.96), non-bacteriliferous males (F = 1.56; d.f. = 1, 9; *p* = 0.24) and bacteriliferous males (F = 0.03; d.f. = 1, 9; *p* = 0.87) had no preference for either treatment (Figure 1). There was no difference in oviposition on leaves between “mock-inoculated” and asymptomatic MBSP-infected plants for non-bacteriliferous (*t* = −0.17; d.f.= 28; *p* = 0.86) and bacteriliferous females (*t* = 1.28; d.f. = 26; *p* = 0.21) (Figure 2A,B).

### 3.2. Preference of D. maidis for Healthy vs. Symptomatic MBSP-Infected Maize Leaves

**Figure 3:**
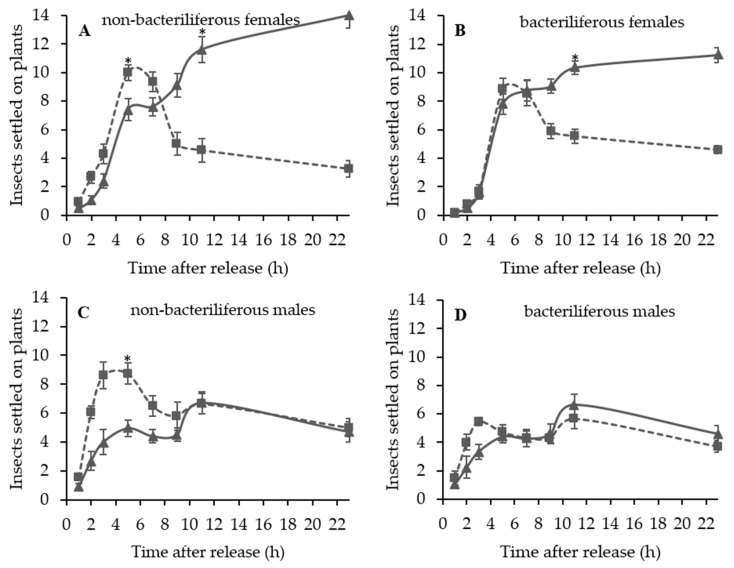
Average number (±SEM) of *Dalbulus maidis* adults on leaves of healthy “mock-inoculated” (solid line) vs. maize bushy stunt phytoplasma (MBSP)-infected symptomatic (dotted line) maize plants, at successive time periods after insect release in the choice arena. (**A**) Non-bacteriliferous females; (**B**) Bacteriliferous females; (**C**) Non-bacteriliferous males; (**D**) Bacteriliferous males. Means at 5 and 9 h after release were compared by *t*-test and the asterisk (*) represents statistical difference (*p* < 0.05).

In the symptomatic stage of the disease, non-bacteriliferous males (*t* = −4.24, d.f. = 18, *p* < 0.001) and females (*t* = −2.71, d.f. = 18, *p* = 0.014) preferred to land on symptomatic leaves in the first 6 h after release in the choice arena (Figure 3A,C). Later, there was a shift in behavior: non-bacteriliferous males (*t* = −1.24, d.f. = 18, *p* = 0.221) were equally distributed among symptomatic and healthy (“mock-inoculated”) leaves (F = 7.29; d.f. = 1, 9; *p* = 0.024) (Figure 3C) and non-bacteriliferous females showed a preference for healthy leaves after 9 h (*t* = 3.60, d.f. = 18, *p* = 0.002) from the beginning of the experiment (F = 10.87; d.f. = 1, 9; *p* = 0.009) (Figure 3A). Bacteriliferous females did not show preference for either treatment (“mock-inoculated” vs. symptomatic) in the first five evaluations after release (*t* = −1.01, d.f. = 18, *p* = 0.326), but after 9 h they clearly preferred healthy leaves (*t* = 4.35, d.f. = 18, *p* < 0.001) (Figure 3B). The bacteriliferous males were equally distributed among healthy and symptomatic leaves, showing no preference for any treatment (Figure 3D). The oviposition of non-bacteriliferous (U = 0.000; *p* < 0.001) and bacteriliferous (U = 0.000; *p* < 0.001) females occurred almost entirely on “mock-inoculated” leaves (Figure 2C,D).

### 3.3. Adults of D. maidis Acquire and Transmit MBSP after 6 h AAP on Symptomatic Plants

In the acquisition efficiency experiment on symptomatic leaves, MBSP was detected in 6.67% (*n* = 30) of females and in 3.33% (*n* = 30) of males, totaling 5% of all insects tested by PCR for the presence of the phytoplasma. Of the six plants inoculated by these insects, 50% were diagnosed with MBSP symptoms.

## 4. Discussion

The success of phytopathogen dissemination by insect vectors depends, among other things, on the host selection behavior of the vector, from the flight orientation to the plant to its acceptance for feeding and/or oviposition [1,2]. Studies have shown that host selection behavior can be influenced by the infection of the host plant by the vector-borne plant pathogen, with implications for pathogen spread [25,26]. In the present study, we showed that maize infection stage (asymptomatic and symptomatic) by MBSP, as well as the infectivity status of the leafhopper vector, *D. maidis*, induced different host selection behaviors.

The observed differences in alighting, settling and oviposition preferences of *D. maidis* on leaves for maize plants at distinct MBSP infection stages (symptomatic and asymptomatic) might be explained by the infection dynamics of the phytoplasma in the plant, which influences visual, olfactory and gustatory cues [27,28]. Because of the differential response of bacteriliferous and non-bacteriliferous leafhoppers, it is also possible that the colonization of the vector’s body by the phytoplasma and/or the previous exposure to symptomatic plants during the immature stage may trigger the changes in the vector’s host selection behavior, as observed for some plant viruses [29]. Different behaviors were also observed between sexes, probably due to the different roles played in the reproductive process, since females need to ensure an adequate plant for offspring, while for males, only for their own nutrition.

In the asymptomatic stage of maize infection by MBSP, bacteriliferous and non-bacteriliferous males, as well as non-bacteriliferous females, were equally distributed on leaves of healthy (“mock-inoculated”) and infected plants. Only bacteriliferous females preferred leaves of mock-inoculated plants, a behavior that is consistent with the “vector manipulation” hypothesis, since it maximizes the chances of transmission, as already proposed for viruses [26] and bacteria [30]. By avoiding diseased plants, bacteriliferous females can inoculate a larger number of healthy plants, generating new infected plants in the field. This behavior can increase the rate of infections of MBSP during the process of primary spread of the disease, when bacteriliferous insects from outside the crop spread the pathogen within the crop, and also of secondary spread, when the acquisition occurs in infected plants within the crop [31,32]. The preference of bacteriliferous females for a healthy (mock-inoculated) plant should allow not only MBSP inoculation but also oviposition on the same plant, with a consequent increase in the natural infectivity of the leafhoppers, since the progeny will develop on asymptomatic plants and likely will acquire the phytoplasma.

During the symptomatic stage of maize infection by MBSP, distinct settling preferences were observed between non-bacteriliferous males and females. Non-bacteriliferous males and females initially landed preferentially on symptomatic plants in the first hours after released in the choice arena, but most females subsequently abandoned the diseased plants and moved to the healthy mock-inoculated plants, whereas the males tended to be equally distributed on the plants regardless of the infections status. On the other hand, bacteriliferous males did not differentiate between mock-inoculated and symptomatic plants throughout the experiment, while bacteriliferous females preferred the mock inoculated plants after an initial period of 5–7 h, during which they did not discriminate between the two treatments. This behavioral shift in the settling behavior of *D. maidis* females when exposed to symptomatic MBSP-infected maize plants is consistent with the “vector manipulation” hypothesis [6] and probably contributes to a faster rate of spread of the phytoplasma in maize crops. The behavior of the non-bacteriliferous *D. maidis* females found in this study is similar to that reported for leafhopper vectors in other pathosystems, e.g., citrus variegated chlorosis and rice tungro disease [30,33]; however, in those studies, unsexed insects were used in the choice assays. A similar shift in vector behavior that possibly favors disease spread was observed for the Asian citrus psyllid, *Diaphorina citri* Kuwayama (Hemiptera: Liviidae), when they were exposed to citrus plants infected with *Candidatus* Liberibacter asiaticus, a bacterium causing citrus huanglonbing [34].

In a previous study with *D. maidis*, it was observed that, in the presence of healthy and MBSP-infected plants, non-bacteriliferous insects avoided landing and ovipositing on infected plants with advanced disease symptoms after 48 h from the beginning of the experiment [20], which corroborates the results of the present study. Interestingly, during the asymptomatic stage of maize infection by MBSP, Gárcia-Gonzales et al. [20] noted a preference of non-bacteriliferous insects for infected plants. However, their preference assays were performed with unsexed insects and by offering the entire plant to the leafhoppers, a condition that differed from the present study, in which we offered only the leaves.

This is the first study that reports differences in behavior between males and females of *D. maidis* regarding host plant selection behavior when exposed to healthy and diseased maize plants. It may be explained by the fact that females tend to settle on plants that are nutritionally more adequate for oviposition and progeny development, whereas males are less selective regarding host nutritional status and tend to move more often among plants, searching for females for mating [35,36].

Considering that non-bacteriliferous females preferably land on plants showing symptoms of the disease in the first 5–7 h of exposure, we investigated whether the leafhoppers could acquire the phytoplasma during that time period. By allowing a 6-h AAP on symptomatic MBSP-infected maize plants, we found that 3.3 and 6.7% of males and females were PCR-positive and that transmission to test plants occurred after a latent period. These results show that *D. maidis* females are able to acquire the phloem-restricted MBSP during the relatively short period (6 h) that they remain on symptomatic plants, and can transmit this pathogen when they move to healthy plants. A study of stylet penetration using the electrical penetration graph (EPG) technique showed that 80% of *D. maidis* individuals take about 3 h to ingest phloem sap when placed on maize plants [37]. Legrand and Power [38] reported that the minimum AAP for the transmission of MBSP is 2 h, but higher transmission efficiencies were observed with longer AAPs.

Regarding the host selection behavior of *D. maidis*, it is possible that the insect initially selects plants influenced by visual and olfactory cues (“orientation preference”) and later, when in contact with the plant, by gustatory cues (“feeding preference”). According to the model proposed by Sisterson [39], if the “orientation preference” guides the vector towards infected plants and the number of healthy plants is higher, the rate of the spread of the pathogen increases; in contrast, if the number of infected plants is greater, the rate of pathogen spread decreases. However, we observed that the acquisition of MBSP by *D. maidis* induces changes in this vector preference, favoring the movement of females towards healthy maize plants after a few hours on diseased plants, which should minimize the importance of the proportion of healthy and infected plants in the rate of spread of MBSP.

The display of symptoms by MBSP-infected maize triggers a “pull–push” strategy, in which the insect vector is stimulated to land on the infected plant, but that plant may have phagodeterrent stimuli that prevent sustained feeding [8,40]. This strategy is also observed in non-persistently transmitted viruses [7,8,33] and in the phloem-limited bacterium *Candidatus* L. asiaticus; in the latter case, however, the psyllid vector remains on the infected plant for a much longer period (days) compared to the aphid vectors of non-persistently transmission viruses, which abandon the infected plant in a few minutes [7,8,34,41]. This “pull–push” strategy in the symptomatic stage of the disease might have an impact on the secondary spread of MBSP. The display of disease symptoms should promote an increase in the number of visits of non-bacteriliferous leafhoppers on infected maize, favoring acquisition and consequently the secondary spread of the phytoplasma in the crop.

Considering that *D. maidis* invades the crop in the early stages of maize development and that inoculation in the later stages may have little effect on yield, primary spread seems to have a much more significant impact in regions where maize is grown in a single season per year [42]. Therefore, vector control in the early stages of the crop is essential to reduce yield losses caused by the disease. However, in regions where maize is grown at different times of the year, symptomatic MBSP-infected plants in an older crop may serve as inoculum sources for subsequent or overlapping plantings. In such cases, in addition to vector control, it is essential to avoid staggered plantings.

## 5. Conclusions

This study shows that MBSP induces shifts in host plant preference of the leafhopper vector, *D. maidis,* that are favorable to its primary and secondary spread. The host selection behavior is influenced by maize infection status and symptom expression, as well as by the leafhopper gender and infection with MBSP. At the early stages of the crop, bacteriliferous females prefer to land and settle on healthy leaves than on leaves of asymptomatic infected plants, a behavior that favors primary spread. As the crop develops and infected plants become symptomatic, non-bacteriliferous males and females initially prefer to land on leaves of infected plants, but a few hours later, the females tend to move towards healthy leaves, a behavioral shift that should increase secondary spread. It remains to be determined whether the observed effects on host selection by bacteriliferous females during the asymptomatic stage of the disease are directly related to vector infection by MBSP or result from a combination of direct and indirect (e.g., development on infected source plants for pathogen acquisition) factors.

## Supplementary Materials

The following are available online at https://www.mdpi.com/2075-4450/11/9/600/s1, Figure S1: Free-choice arena 30 × 30 × 30 cm. A—Side view. B—Side view with (a) side opening (4 × 20 cm) sealed with “voile” fabric for ventilation. C—Top view with (b) openings (4 × 20 cm) for ventilation and (c) “Falcon” tube from which the insects were released. D—Internal side view showing the openings through which the leaves were inserted (d) 1.5 × 6.0 cm (e) 1.5 × 7.0 cm.
