# Supplementary figures and images for: Maize Bushy Stunt Phytoplasma Favors Its Spread by Changing Host Preference of the Insect Vector
Source: Insects. 2020 Sep 5;11(9):600. doi: 10.3390/insects11090600 (PMC7565095; doi:10.3390/insects11090600)

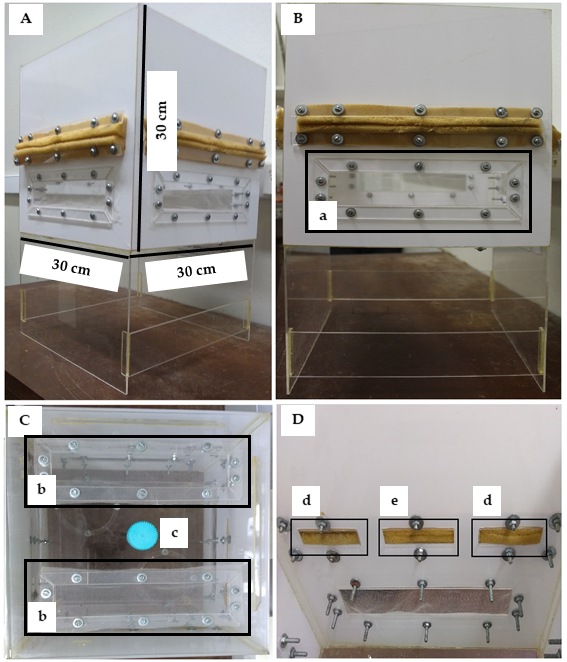

Supplement: Supplementary file 1 [file insects-11-00600-s001.zip › insects-905303-supplementary.tif]
